# Commentary on: “Detection of *Toxoplasma gondii* in raw caprine, ovine, buffalo, bovine, and camel milk using cell cultivation, cat bioassay, capture ELISA, and PCR methods in Iran”

**DOI:** 10.3389/fmicb.2015.00215

**Published:** 2015-03-18

**Authors:** Sonia Boughattas

**Affiliations:** Department of Environmental and Biological Chemistry, College of Agriculture, Life and Environmental Sciences, Chungbuk National UniversityCheongju, South Korea

**Keywords:** Toxoplasma, infection, foodborne, milk, consumption

As it is known, toxoplamsosis occurs mainly by foodborne transmission: ingestion of raw or undercooked meat; unwashed fruit/vegetables, unhygienic water, or contaminated milk. Gaps in the concerning risk assessment of *Toxoplasma gondii* (T. gondii) by milk consumption are noted.

Recently, a paper launched a debate about milk contamination (Dehkordi et al., 2013). The authors reported the detection of the toxoplasmic DNA in milk of different naturally infected herds (ovine, caprine) including low sensitive hosts to the parasite (bovine, camels, buffalo). American scientists (Dubey and Jones, 2014) commented the findings pointing out some technical gaps about the sensitivity of employed methods (e.g., high rate of parasite detection by bioassay and cell culture), rising thus interrogations and seeking independent reproduction of data to affirm the reported conclusion.

Risk assessment studies associating *T. gondii* infections and milk consumption showed wide divergence. While some papers reported positive correlations between drinking milk and humans infection in Poland (Paul, 1998), USA (Jones et al., 2009), Mexico (Alvarado-Esquivel et al., 2010); other studies stated non-significant influence of milk or milk products consumption among pregnant women in Jordan (Nimri et al., 2004) and high risked populations in Kyrgyzstan (Minbaeva et al., 2013).

Essentially, outbreaks of human toxoplasmosis were reported mainly by the ingestion of raw (unpasteurized) goat milk with the contamination of 10 members from 24 individuals composed family. The authors stated serological evidence of acute *T. gondii* infection (One subject with retinochoroiditis and the other 9 persons had asymptomatic infections). All 10 seropositive persons had recently consumed raw goat's milk from the family herd as compared with no consumption of raw milk by the 14 persons with negative results. No dietary item or other risk factors were as strongly associated with positive serological test results as raw milk consumption (Sacks et al., 1982). Studies worldwide attempted to analyze the risk factor of raw goat milk consumption among different populations: in Brazil when targeting pregnant women (Avelino et al., 2003; Barbosa et al., 2009; Moura et al., 2013); in Saudi Arabia targeting the same population (Almushait et al., 2014); in USA questioning a global population (Jones et al., 2009); in Mexico focusing on agricultural workers (Alvarado-Esquivel et al., 2013) and in Ethiopia investigating women population in general (Gebremedhin et al., 2013).

It has often been thought that the risk of acquiring an infection with *T. gondii* by drinking goat's milk is high and cow's milk, if any, is minimal. However, it was shown that only 1.6% of the control patients in USA specified they drank unpasteurized goat's milk in the past 12 months (Jones et al., 2009). Consequently consumption of unpasteurized goat's milk is retained relatively uncommon in the United States. Lately it was found that 14.1% of the seropositive pregnant women in Brazil had the habit of consuming raw cow's or/and goat's milk (Moura et al., 2013). When risk factors analyzed, unpasteurized/raw cow's milk consumption wasn't associated with toxoplasmic infection among agricultural workers in Mexico (Alvarado-Esquivel et al., 2013) as well in Brazil when targeting immune-competent population (Bahia-Oliveira et al., 2003). However other studies reported significant association between milk/dairy consumption and toxoplasmosis occurrence: in Brazil (Heukelbach et al., 2007; Santos et al., 2009; Silva et al., 2014); in Mexico (Alvarado-Esquivel et al., 2010); in Egypt (Elsheikha et al., 2009) and in Iran (Fouladvand et al., 2010).

Positive correlation was also retained with the consumption of other animals' milk. Indeed, risk factors analysis reported the consumption of raw buffalo dairy products in British Columbia (Proctor and Banerjee, 1994) and in Egypt among blood donors (Elsheikha et al., 2009). Sheep milk involvement in toxoplasmosis occurrence was also investigated as potential risk factors in Ethiopia (Gebremedhin et al., 2013) when targeting women population as well as in Brazil (Barbosa et al., 2009) when targeting pregnant females.

Few other studies investigated the detection of natural infection of the parasite within less consumed milk from camel and donkeys. In Iran, scientists reported a rate of camel milk contamination of 3.12% by culture bioassays (Dehkordi et al., 2013). In the frame of seroepidemiological investigation within camels, Ethiopian team revealed that 100% of the animal owners had consumed raw camel milk (Gebremedhin et al., 2014). Concerning *T. gondii* prevalence in the milk matrix of donkeys, only three studies investigated its analysis. Scientists in Egypt were able to detect the toxoplasmic antibodies in 46.3% of milk samples (Haridy et al., 2010). In Europe, Italian studies using molecular tools reported the detection of *T. gondii* DNA in 66.66% (Mancianti et al., 2014) and in 22.22% (Martini et al., 2014) of analyzed donkeys' milk samples.

The toxoplasmic transmission was attributed both to tachyzoites in the milk and to suckling. Toxoplasmosis was described in breast fed child whose mother had recently acquired toxoplasmosis (Azab et al., 1992; Bonametti et al., 1997). Moreover, mammary glands can be contaminated from environment and suckling calf-camel can acquire toxoplasmosis from milk/nipples of their infected mother. The toxoplasmic contamination is worsened due to smaller concentration of proteolytic enzymes that counter the parasite infection in the intestine of children and suckling animals (Ishag et al., 2006). It was experimentally confirmed that tachyzoites survived in the milk for three to 7 days at 4°C (Spišák et al., 2010) and in homemade fresh cheese for a period of 10 days (Hiramoto et al., 2001), proving consequently that raw milk can serve as a source of *T.gondii* infection.

Proponents of raw milk claim that unpasteurized milk/dairy products are more nutritive than pasteurized ones, even if it is stated elsewhere with equal nutritional values (Claeys et al., 2013). It was reported that many Americans consume products labeled as “organic” or from food cooperatives (Dubey and Jones, 2014). In Europe and according to the regional legislations; raw milk from any species can be sold immediately after milking by the producer or a local milk seller to the consumer, without any thermal treatment except refrigeration between 0 and 4°C (Mancianti et al., 2014).

Given that, the increase of organic milk demand, and in the light of these recent observations, there is great concern regarding whether it is safe to consume raw milk. Deeper analysis should be investigated and special awareness should be taken by consumers with the insistence on of heat treatment of the milk before consumption.

## Conflict of interest statement

The author declares that the research was conducted in the absence of any commercial or financial relationships that could be construed as a potential conflict of interest.
